# Dissecting CYP1A2 Activation by Arylalkanoic Acid Prodrugs toward the Development of Anti-Inflammatory Agents

**DOI:** 10.3390/ijms25010435

**Published:** 2023-12-28

**Authors:** Maria Antonietta Occhiuzzi, Giuseppina Ioele, Michele De Luca, Bruno Rizzuti, Domenica Scordamaglia, Rosamaria Lappano, Marcello Maggiolini, Antonio Garofalo, Fedora Grande

**Affiliations:** 1 Department of Pharmacy, Health and Nutritional Sciences, University of Calabria, 87036 Rende, CS, Italy; mariaantonietta.occhiuzzi@unical.it (M.A.O.); giuseppina.ioele@unical.it (G.I.); michele.deluca@unical.it (M.D.L.); domenica.scordamaglia@unical.it (D.S.); rosamaria.lappano@unical.it (R.L.); marcello.maggiolini@unical.it (M.M.); antonio.garofalo@unical.it (A.G.); 2 CNR-NANOTEC, SS Rende (CS), Department of Physics, University of Calabria, Via Pietro Bucci, 87036 Rende, CS, Italy; 3 Institute of Biocomputation and Physics of Complex Systems (BIFI), Joint Unit GBsC-CSIC-BIFI, University of Zaragoza, 50018 Zaragoza, Spain

**Keywords:** NSAIDs, metabolism, drug/enzyme interaction, COX inhibition, molecular docking

## Abstract

Arylalkane-derived prodrugs of arylacetic acids are a small group of substances that have long been known for their anti-inflammatory action. Despite their ease of synthesis and good potential for the development of new potent and safe anti-inflammatory agents, this group of substances has not received much attention from researchers so far. Therefore, representative arylalkane derivatives were investigated through molecular docking techniques to verify the possible hepatic activation mode toward active metabolites by CYP1A2. In this regard, arylalkanoic acid prodrugs were docked with a crystallographic structure of human CYP1A2, in which the enzyme is co-crystallized with the selective competitive inhibitor α-naphthoflavone BHF. Of note, all the examined compounds proved capable of interacting with the enzyme active site in a manner similar to Nabumetone, thus confirming that a productive metabolic transformation is feasible. On the basis of these findings, it is possible to argue that subtle differences in the way CYP1A2 accommodates the ligands depend on the fine details of their molecular structures. Overall, these data suggest that compounds simply formed by an aromatic moiety bearing an appropriate alkane-derived chain could lead to innovative anti-inflammatory agents.

## 1. Introduction

**Figure 1:**
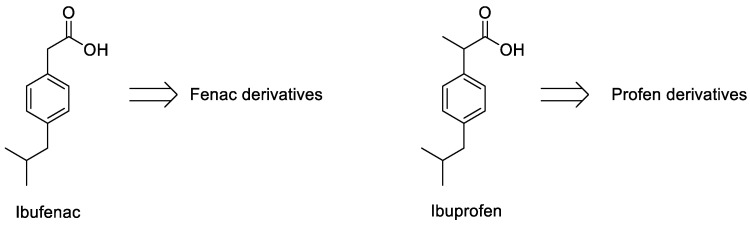
Reference drugs for Fenac and Profen derivatives.

The arylalkanoic acid derivatives represent the largest class of NSAIDs (non-steroidal anti-inflammatory drugs). Aryl and heteraryl acetic acids (termed Fenac, all considered derivatives of Ibufenac), as well as 2-aryl and 2-heteraryl propionic acids (Profen and Ibuprofen derivatives), are the most popular subclasses of the group (Figure 1). Similar to the anthranilic acid and oxicam derivatives, all of them inhibit with different selectivity the two isoforms of the cyclooxygenase enzyme, COX-1 and COX-2, by peripherally limiting prostaglandin biosynthesis. This mechanism of action explains, at least in part, their anti-inflammatory efficacy as well as their main intrinsic side effect, i.e., gastrointestinal irritation [1]. Several drugs belonging to this class are widely used for the long-term treatment of autoimmune inflammatory diseases [2]. Furthermore, different NSAIDs, in particular COX-2-selective agents, could play a key role in the prophylaxis or adjuvant treatment of different types of cancer [3]. NSAIDs are generally characterized by rapid achievement of peak plasma concentration, high plasma protein binding, and low distribution volume after oral administration. The plasma disappearance half-life is often a few hours, while longer times can be reached with the use of prodrugs, which require hepatic activation and allow for once-daily dosing [4].

The prodrug strategy in the oral treatment of inflammation is successfully adopted to attain therapeutic improvements, such as a reduction of ulcerogenic effects in part attributed to the administration of acidic molecules. In fact, although the systemic ulcerogenic action due to the limitation of prostaglandin production remains inevitable, the containment of back-diffusion of H^+^ in the gastric mucosal cells could help to limit this side effect [5].

In this context, the putative metabolic activation of classic arylalkane prodrugs has been investigated by molecular docking to the active site of CYP1A2. The aim of our simulations is to demonstrate the ability of such compounds to interact with this molecular target by anchoring in the catalytic site with favorable binding energy, which is a necessary step towards a metabolic conversion into active molecules. The results contribute to unveiling the key molecular determinants leading to association with CYP1A2, clarifying the interplay between the protein residues most involved in the binding and the aromatic and alkane-derived chain moieties that characterize the scaffold of the ligands.

## 2. Results and Discussion

### 2.1. Arylakanoic Acid Prodrugs

**Figure 2:**
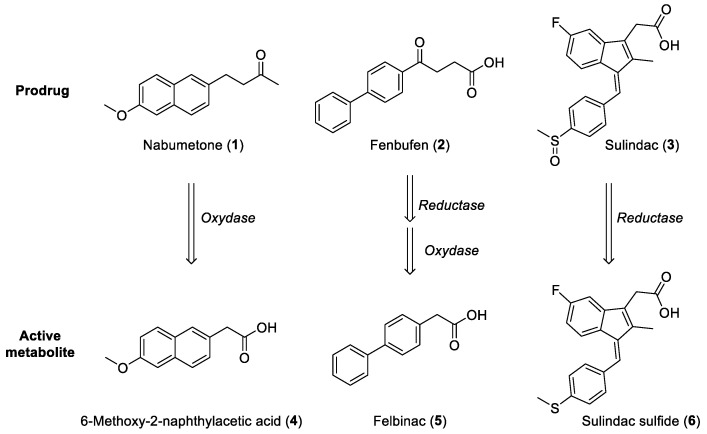
NSAID prodrugs and corresponding active metabolites.

A total half-life of over 22 h was observed for Nabumetone (**1**), 10 h for Fenbufen (**2**), and 16 h for Sulindac (**3**), representing three relevant examples of prodrugs, and these values always refer to the corresponding active metabolites 6-methoxy-2-naphthylacetic acid (**4**), 2-([1,1′-biphenyl]-4-yl)acetic acid (Felbinac, **5**) and Sulindac sulfide (**6**), respectively (Figure 2).

Prodrugs obtained by simply derivatizing the carboxylic moiety of the active molecule by transforming it into an ester or amide, or by using buffered pharmaceutical forms, have been extensively studied for salicylates. However, this strategy usually does not lead to satisfactory containment of the ulcerogenic effects, probably due to a fast restoration of the acidic function by hydrolases still within the gastrointestinal mucosa. Better results have been obtained when the activation of a molecule, initially lacking acidic function, takes place in the liver, where more complex metabolic transformations somehow generate derivatives, generally containing an acetic moiety, which is crucial for interaction with COX. This is the case of the prescription drug Nabumetone (**1**), an inactive neutral ketone rapidly converted in the liver to 6-methoxy-2-naphthylacetic acid (**4**), a metabolite with potent COX inhibitory activity and an overall low incidence of ulcerogenic action. Compound **4**, like all Fenac and Profen, therefore undergoes glucuronidation prior to renal excretion [6]. On the other hand, the derivatives that carry side chains that are still acidic but bulkier than acetic or 2-propionic ones generally show a longer duration of action due to the need to be metabolically activated by chain-shortening enzymatic transformation. Fenbufen (**2**), a derivative of 4-oxobutanoic acid, represents the prototype of such a class of prodrugs as its activation takes place after hepatic transformation into the corresponding acetic derivative Felbinac (**5**) [7]. Several analogues of Fenbufen possess alternative biological activities, such as choleretic, cholagogue, and muscle relaxant effects, on the gastrointestinal tract, plausibly attributable to metabolites formed after processes similar to those proposed for the parent drug [8].

**Figure 3:**
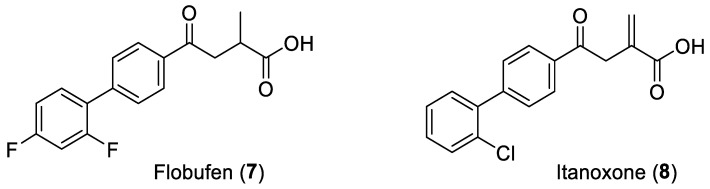
Chemical structure of Flobufen (**7**) and Itanoxone (**8**).

Flobufen (**7**), an aryl-4-oxo-2-methylbutanoic acid (Figure 3), can be considered a homologous of the previous derivatives and appears to undergo a similar side chain biotransformation to the corresponding Fenac endowed with anti-rheumatic properties [9]. It has been shown that the close analogue Itanoxone (4-(2′-chloro-[1,1′-biphenyl]-4-yl)-2-methylene-4-oxobutanoic acid, **8**), is able to generate the corresponding Fenac in vivo after a metabolic pathway including the carbonyl reduction and the shift of the methylene double bond along the side chain before the chain shortening step. A pronounced lipid-lowering effect was found for this compound, together with moderate anti-inflammatory activity, likely attributable to the metabolite [10].

**Figure 4:**
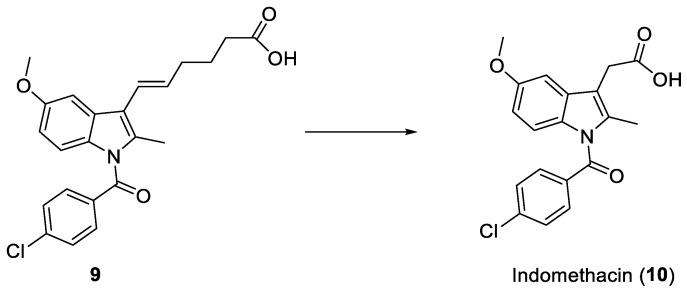
Metabolic transformation of prodrug **9** to Indomethacin (**10**).

A metabolic activation similar to that of 4-oxobutanoic acids has also been attempted for hex-5-enoic acid derivatives. In particular, the conversion of (*E*)-6-(1-(4-chlorobenzoyl)-5-methoxy-2-methyl-1*H*-indol-3-yl)hex-5-enoic acid (**9**) to Indomethacin (**10**) was studied in vivo (Figure 4). Thus, a process was demonstrated that involves a sequential double β-oxydation on the side chain of the prodrug, restoring the initial form of the potent anti-inflammatory agent and prolonging its activity up to 24 h [11].

**Figure 5:**
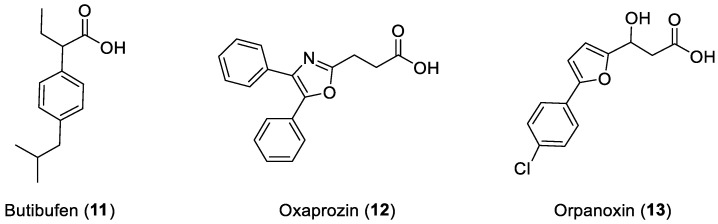
2-Arylbutanoic and 3-arylpropanoic COX inhibitors.

The presence of an acetic or 2-propionic moiety in the structure seems mandatory for the activity of all these compounds on COX. However, a few examples of active compounds containing alternative groups are present within the anti-inflammatory armamentarium. The 2-arylbutanoic acid Butibufen (**11**) and the 3-arylpropanoic acids Oxaprozin (**12**) and Orpanoxin (**13**) are agents capable of interacting with COX without undergoing previous metabolic transformation (Figure 5) [12].

Despite these overall results, studies on most of the described compounds have not been continuous over time. However, the good potential of the above-described prodrugs, combined with the relative ease of synthesis for obtaining new derivatives, would allow a broad development of research aimed at identifying new effective and safe anti-inflammatory agents. In order to obtain more detailed information and find strategies for obtaining new analogues, all the above NSAID precursors were docked within the CYP1A2 catalytic site to test the binding mode for productive conversion into active agents.

### 2.2. The Binding Site of CYP1A2

**Figure 6:**
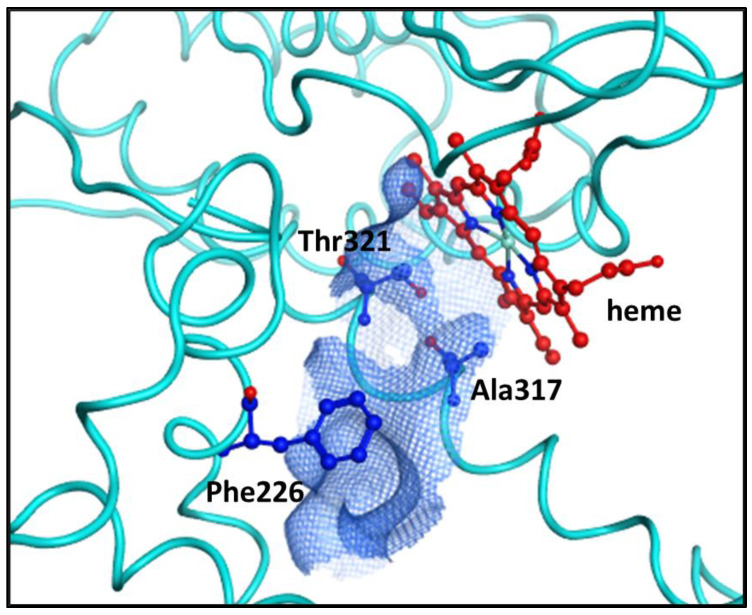
Schematic representation of the CYP1A2 binding site (blue grid) in close proximity to the heme co-factor.

The docking experiments were carried out using the 2HI4 crystallographic structure of human CYP1A2, the sole complex present in the Protein Data Bank (PDB), in which the enzyme is co-crystallized with the α-naphthoflavone BHF (2-phenyl-4*H*-benzo[*h*]chromen-4-one), which behaves as a selective competitive inhibitor. This structure, successfully employed for the study of several ligands of this enzyme [13,14], constitutes 495 amino acid residues forming 12 α-helixes and 4 β-sheets. The binding site is delimited by two-folded α-helixes and the heme prosthetic group, resulting in a cavity able to host planar compounds like BHF. Within the cavity, on the side opposite to heme, the presence of a Phe226 residue contributes to conferring a narrow and extended shape to the entire binding pocket (Figure 6). Together with Phe226, other key protein residues that assist the association of molecular partners are Ala317 and Thr321, which favor the binding affinity by providing further hydrophobic and π-stacking interactions. Due to its peculiar architecture, distinct from other CYP subfamilies, CYP1A2 appears to have evolved for the specific oxidation reaction on substituted aromatic compounds [15].

However, this enzyme has been recognized as the main reason for the metabolic activation of Nabumetone (**1**) by catalyzing a sequence of reactions involving its alkanone moiety. It was hypothesized that the same enzyme would act with a similar mechanism on compounds structurally related to **1** [16]. All the known compounds having significance in the anti-inflammatory field and showing an arylalkanoic structural motif have been herein selected for performing an in silico investigation in order to establish whether a coherent metabolic activation by CYP1A2 could be hypothesized.

### 2.3. Nabumetone

Nabumetone (**1**) was first described and patented, together with several congeners, by a British company in 1977, and it remains the most relevant example of an anti-inflammatory arylalkanoic acid precursor that entered the market [17,18]. The comparison with known analgesics such as aspirin, indomethacin, and naproxen clearly showed remarkable activity with a lower incidence of gastrointestinal side effects. This apparent lower gastrolesivity is still debated, although it seems that even the active metabolite 6-methoxy-2-naphthylacetic acid (**4**) is less toxic than naproxen and other NSAIDs. It is worth noting that about 35% of a 1000 mg dose is converted to **4**, while 50% of the drug is converted to unidentified metabolites. Compound **4** is in turn biotransformed in the liver by CYP2C9 to an inactive metabolite, which is partly eliminated unchanged and partly as a conjugate. This particular pharmacokinetics does not allow this acid to enter the enterohepatic circulation, and this would contribute to the lack of severe irritation on the gastrointestinal tract. Nabumetone (**1**) contains no acidic functionality and passes through the stomach without producing irritation. Moreover, no significant contribution of the microbiota to Nabumetone (**1**) metabolism in the gut was yet detected, although a residual amount of the corresponding alcohol resulting from the reduction of the keto group by intestinal bacteria could not be excluded [19].

**Figure 7:**
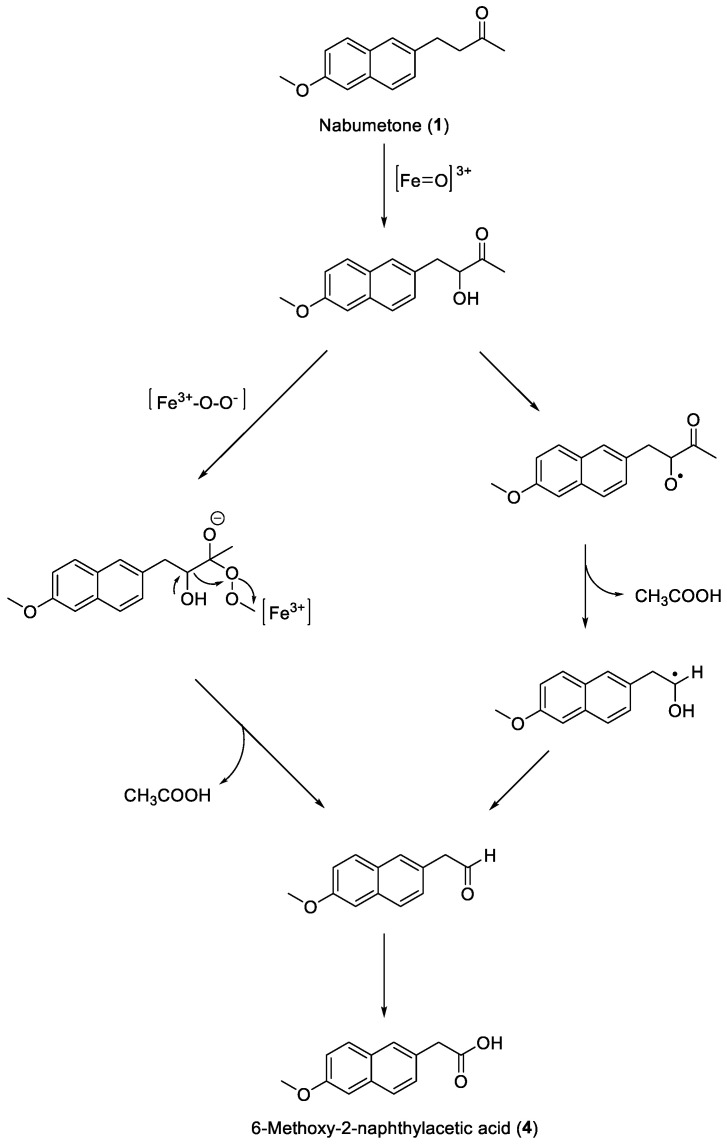
Hypothetical mechanisms for the CYP1A2-catalyzed metabolism of Nabumetone (**1**).

Compound **1** (Figure 2) remains among the few analogues subjected to extensive metabolic studies. Although the formation of the active metabolite **4** has been conclusively ascertained, the overall pathway through which this occurs has long remained unclear. The active metabolite **4** was found to be active on both COX isoforms (COX-1 and COX-2), with a marked preference for COX-2 [20]. Indeed, the conversion must necessarily take place through a carbon–carbon bond cleavage, which is known to be a rather rare reaction occurring during mammalian metabolism. However, a predominant role of CYP1A2 in the catalysis of the reaction has been hypothesized, and consequently, a plausible and comprehensive mechanism has been proposed. With the help of potential intermediates suitably synthesized and used as substrates, it has been possible to study in depth the role played by CYP1A2 and other enzyme isoforms during the conversion of **1** to **4**. Three main steps should be included in the entire process: (1) 3-hydroxylation of Nabumetone (**1**) by a normal P450 ferryl species ([Fe=O]^3+^); (2) carbon–carbon bond cleavage of 3-hydroxynabumetone to 2-(6-methoxynaphthalen-2-yl)acetaldehyde promoted by a ferric peroxo anion ([Fe^3+^-O-O^−^]); (3) oxidation of the aldehyde to 6-methoxy-2-naphthylacetic acid (**4**) again by [Fe=O]^3+^ (Figure 7, left). This overall pathway is accounted for by the presence of the carbonyl group of **1**, which assists the enzyme in initially promoting hydroxylation at C3 on the side chain [16]. An alternative mechanism involving a free-radical-mediated reaction was, however, proposed. This process would start with the C3 alcohol formation followed by the hydroxyl hydrogen abstraction to give an alkoxy radical, which would generate a carbon radical and acetic acid. The transfer of an electron to an iron-oxygen complex generates an aldehyde, which subsequently oxidizes to the corresponding acid (Figure 7, right). However, the latter mechanism, which excludes the intervention of species other than [Fe=O]^3+^, has not been conclusively demonstrated [21].

**Figure 8:**
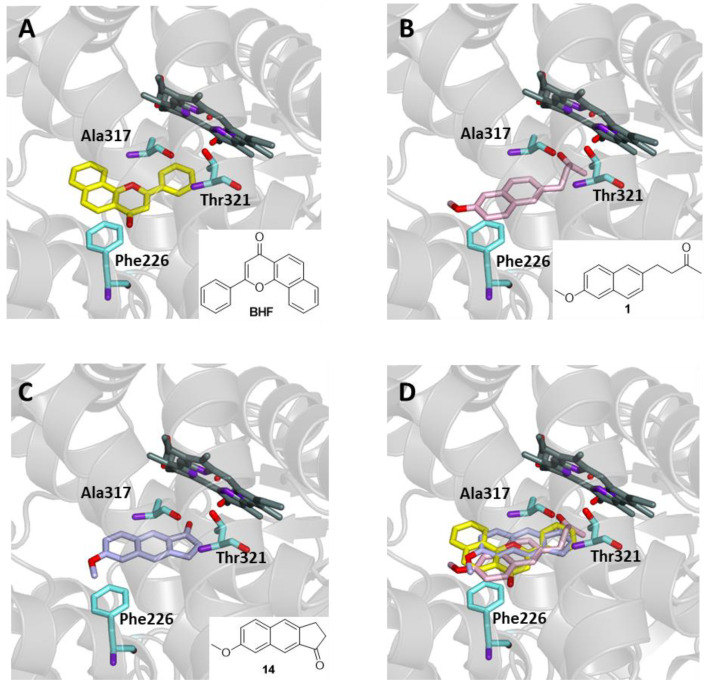
Ligand-binding pocket of the active site of CYP1A2; ribbons representing protein structural elements are shown. The key residues are also indicated. (**A**) Binding mode of the crystallographic ligand BHF (yellow); (**B**) binding mode of Nabumetone (pink); (**C**) binding mode of ketone **14** (purple); (**D**) superimposed binding mode of the three ligands.

A similar process could be envisaged for the metabolic activation of the recently described ketone **14** (Figure 8), a tricyclic analogue of Nabumetone (**1**). Although data on its activity are not yet available, this compound can undergo in vivo activation by sequential oxidation reactions that occur during the cleavage of a single carbon atom from the pentatomic ring [22].

A topical use of gel formulations containing **1** and excipients aimed at facilitating the skin-crossing of the drug has also been hypothesized, although it remains unclear whether biotransformation to **4** can effectively occur in the microsomal fraction of endoplasmic reticulum of tissues other than the liver, where CYPs are still operating [23]. Formulations based on microemulsion delivery systems have been designed for transdermal administration at the joint inflammation site, hypothesizing an extra-hepatic metabolism [24].

In order to verify the capability of Nabumetone (**1**) and the tricyclic analogue **14** to behave as substrates for CYP1A2, the two molecules were docked into the binding site of the enzyme and compared with the known crystallographic ligand BHF. As shown in Figure 8, both compounds are able to enter the binding site of CYP1A2, interacting with key residues in a manner similar to the known ligand, with favorable binding energy values of −9.6 and −10.2 kcal/mol for compounds **1** and **14**, respectively, even though less favorable than BHF (−14.0 kcal/mol). In particular, hydrophobic and π-stacking interactions with Phe226, Ala317, and Thr321 are involved in all cases.

### 2.4. Fenbufen and Analogues

Fenbufen (4-([1,1′-biphenyl]-4-yl)-4-oxobutanoic acid, **2**) and numerous other congeners have been obtained by a smooth reaction between an aromatic derivative and succinic anhydride under Friedel-Crafts conditions. A marked anti-inflammatory activity has been soon associated with this compound, which served as a prototype for the identification of several other analogues. However, despite its good potential as an anti-inflammatory agent, the drug was withdrawn from the market because of its liver toxicity [25]. It should be noted that the major metabolite of Fenbufen (**2**), marketed as Felbinac (**5**, Figure 2), is still used in the topical treatment of traumatic injuries and osteoarthritis [26].

A small library of analogues of **2** has been recently obtained by a Suzuki–Miyara coupling of a boronic acid derivative with a variously substituted bromobenzene. Some of these compounds have shown activity against the release of IL-1 and the ability to considerably inhibit COX-2, with a high degree of selectivity over COX-1, in in vitro anti-enzymatic assays. This latter property was confirmed after docking experiments performed on a crystallographic structure of COX-2 [27].

**Figure 9:**
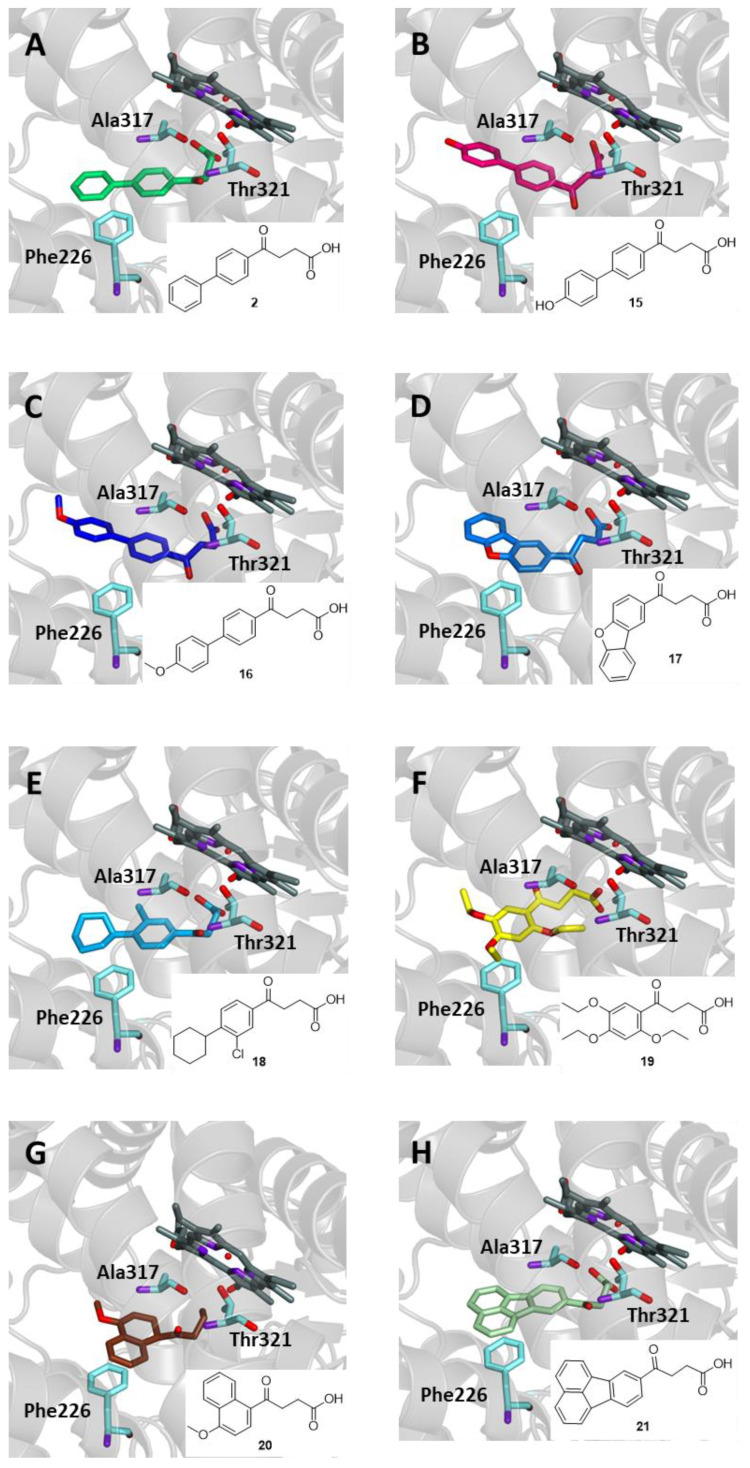
Binding mode in the active site of CYP1A2 of (**A**) Fenbufen (**2**, green); (**B**) *p*-hydroxyfenbufen (**15**, fuchsia); (**C**) *p*-methoxyfenbufen (**16**, violet); (**D**) Furobufen (**17**, blue); (**E**) Bucloxic acid (**18**, cyan); (**F**) Trepibutone (**19**, yellow); (**G**) Menbutone (**20**, brown); (**H**) Florantyrone (**21**, light green).

The most active derivative, *p*-hydroxyfenbufen (**15**, Figure 9), showed potency comparable to that of Celecoxib, one of the most prescribed COX-2 selective inhibitors. The structurally related *p*-methoxyfenbufen (**16**) was previously patented for its inhibitory activity on matrix metalloproteinases, therefore potentially useful for the treatment of numerous pathologies, including inflammation [28].

The 4-oxobutanoic acid derivatives **17–21** also showed some interesting biological properties. A coherent explanation for the metabolism of Fenbufen (**2**) and congeners **15–21**, all of which bear a carbonyl moiety next to the aromatic system, includes a preliminary reduction from ketone to alcohol before the above-seen chain shortening process. In fact, in the case of Fenbufen (**2**), 4-([1,1′-biphenyl]-4-yl)-4-hydroxybutanoic acid along with Felbinac (**5**) have been isolated as the two major metabolites [29,30]. A close analogue of Fenbufen could be considered Furobufen (**17**), in which a dibenzofurane replaces the biphenyl as the aromatic moiety. The compound was synthesized several decades ago and, although it showed a good potential as an anti-inflammatory agent, producing antiarthritic and antipyretic effects together with an analgesic action in inflamed tissues, has never been marketed [31]. It was ascertained that the drug was rapidly converted into 2-(dibenzo[*b*,*d*]furan-2-yl)acetic acid, which represents the major circulating metabolite, either in animals or in humans. The half-life of the metabolite in humans has been calculated to be approximately 20 h [32]. Bucloxic acid (**18**), a cyclohexylphenyl analogue, has been first proposed as a neuromuscular and anti-inflammatory agent for use in chronic glomerular nephropathy. Due to several serious toxic effects, the drug was never introduced to the market but was only used for research purposes [33]. The 4-oxobutanoic derivative Trepibutone (**19**), apparently devoid of any anti-inflammatory activity, has been experimented with as a choleretic agent useful for muscle relaxation by accelerating cellular calcium intake during the treatment of bile duct and pancreatic disease in animals [34]. Theoretical pharmacokinetic studies have been performed, which did not predict any transformation of the alkyl side chain [35]. However, its chemical structure suggests that it may undergo a metabolic transformation similar to that of the parent compounds.

A therapeutic effect similar to Trepibutone has been attributed to Menbutone (genabilic acid, 4-methoxy-γ-oxo-1-naphthalene butanoic acid, **20**), a further derivative of this class widely used in veterinary medicine to treat digestive disorders in various animal species [36]. Although a study has been conducted on its metabolism in sheep, it is unclear whether any hepatic transformation leads to side chain shortening, since a high rate of unchanged drug was recovered after oral administration in rats [37]. The preparation of Florantyrone (**21**), the fluoranthene congener of the previous compounds, has been known for a long time. This compound acts as a cholagogue and choleretic and is used in the treatment of biliary dyskinesia as such or in combination with a bile acid. Florantyrone (**21**) has been shown to be converted into several metabolites in vivo, but no evidence has ever been provided for the formation of the corresponding acetic acid metabolite [38]. Therefore, its possible anti-inflammatory action remains to be ascertained.

This group of 4-oxobutanoic acids was subjected to docking experiments with the CYP1A2 binding site in order to establish whether a conversion into acetic derivatives is possible. As shown in Figure 9, all compounds are able to enter the binding site of CYP1A2, interacting with Phe226, Ala317, and Thr321 residues, as described previously, with binding energy values ranging from −8.4 to −13.2 kcal/mol. Notably, compounds **18**, **20,** and **21** further interact through the carboxyl group with Thr321 by hydrogen bonding, resulting in the best binding energy values (−10.0, −9.7, and −13.2 kcal/mol, respectively).

### 2.5. Flobufen and Analogues

Flobufen (4-(2′,4′-difluorobiphenyl-4-yl)-2-methyl-4-oxobutanoic acid, **22**) was identified as a potent antiarthritic agent with pronounced anti-inflammatory and immunomodulatory effects, along with a rather low toxicity. It was first prepared by using methylsuccinic anhydride in a similar fashion to Fenbufen (**2**), although more modern methods have been recently described for the synthesis of compounds of this type [39,40]. Flobufen (**22**) represents the prototype of compounds that differ from the previous derivatives by the presence of a methyl group on the carbon adjacent to the carboxylic function. This structural feature gives rise to two enantiomeric forms, and this could therefore be reflected in more complicated metabolic pathways, leading to the formation of the corresponding Fenac as a major metabolite endowed with anti-inflammatory activity. The isolation of several metabolite isomers of dihydroflobufen suggests that the metabolic pathway would begin with a reduction of the ketone to alcohol, followed by side chain oxidation, including carbon–carbon bond cleavage, taking up what is above reported in Figure 7. Finally, the active acetic acid metabolite undergoes glucuronidation before renal excretion [41]. Metbufen (**23**) is another anti-inflammatory drug, closely analogue of **22**, from which it differs by the absence of the two fluorine atoms in the structure. This compound has only been used for pharmacokinetics investigation, and a metabolic fate similar to that of the parent compound, leading to an active Fenac, has been hypothesized [42].

A similar metabolic destiny could be envisaged for (1*S*,2*R*)-1-methyl-4-oxo-1,2,3,4-tetrahydronaphthalene-2-carboxylic acid (**24**), a compound tested for antitumor activity, but found inactive, while it has never tested against inflammation. The side chain of this compound forms a cyclic structure, which somehow makes it a conformationally restricted analogue of previous compounds, thus probably susceptible to undergoing a similar metabolic transformation. Although the putative phenylacetic acid that would form after metabolism is of no utility against inflammation, its formation would confirm the character of the CYP1A2 substrate for compound **24** [43]. Itanoxone (**25**) is a drug chemically related to Flobufen as it bears a methylidene instead of a methyl on carbon 2 and a chlorine atom on the 2′-position of the biphenyl. Its hypolipidemic and hypouricemic activity has been attributed to corresponding Fenac generated in vivo. The acidic metabolite, found in crab, rat, and human species, appears to be responsible for the anti-inflammatory activity involving COX inhibition [44]. The compound was able to selectively inhibit both the malondialdehyde platelet enzyme and COX while partially preventing the inhibitory effects of aspirin [42].

**Figure 10:**
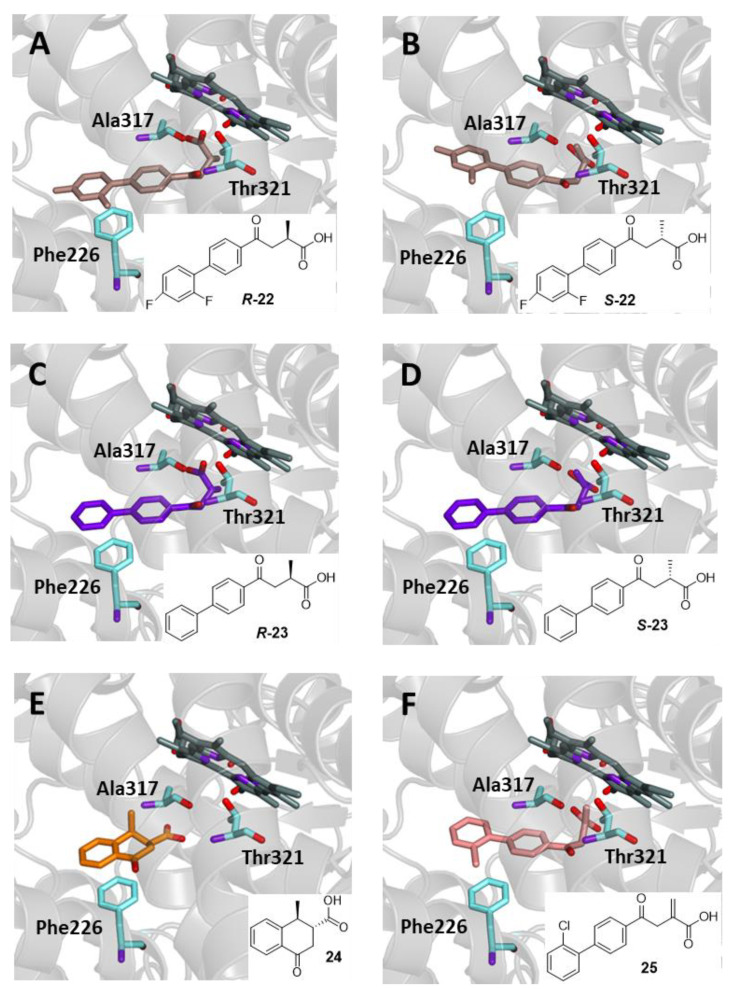
Binding mode in the active site of CYP1A2 of Flobufen (**22**) (**A**) ***R*-22** (brown); (**B**) ***S*-22** (brown); Metbufen (**23**) (**C**) ***R*-23** (violet); (**D**) ***S*-23** (violet); (**E**) compound **24** (gold); (**F**) Itanoxone (**25**, pink).

All acids in this group have been subjected to docking experiments with CYP1A2 as described above, and all were able to enter the binding site of CYP1A2 (Figure 10). Enantiomers of Flobufen (**22**) and Metbufen (**23**) were analyzed individually, and, interestingly, no significant differences in the binding mode were observed, so the catalytic site can accommodate compounds regardless of the absolute configuration. In all cases, hydrophobic interactions with Phe226, Ala317, and Thr321 resulted in a binding affinity of −9.6 and −9.8 kcal/mol, respectively. Compound **24** and Itanoxone (**25**) showed a similar mode of interaction, except for the absence of hydrogen bonding, resulting in binding energy values of −8.7 and −10.2 kcal/mol, respectively.

### 2.6. Hexanoic Acids

**Figure 11:**
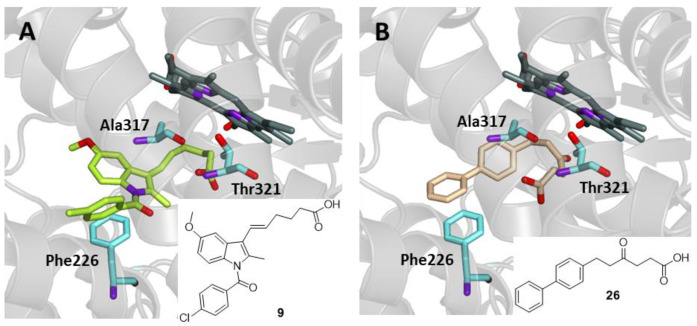
Binding mode in the active site of CYP1A2 of (**A**) compound **9** (green) and (**B**) compound **26** (beige).

Noteworthy activity in an in vivo model of inflammation was recorded for 6-([1,1′-biphenyl]-4-yl)-4-oxohexanoic acid (**26**) and a small series of its derivatives smoothly obtained after an initial acylation between a suitable aromatic aldehyde and levulinic acid. These compounds have been compared to Fenbufen (**2**) for the inhibition of carrageenan-induced rat paw oedema, while having no effect on arachidonic acid metabolism in vitro after a human whole blood assay. This behavior was again explained by hypothesizing the metabolic transformation of these compounds into active short-chain acids, mostly following what was reported above for compound **9** [45]. Due to their structural similarity, compound **9** and the selected representative 6-([1,1′-biphenyl]-4-yl)-4-oxohexanoic acid (**26**) were subjected to docking experiments. The latter compound was chosen within the series due to its structural analogy to Fenbufen (**2**). As shown in Figure 11, both compounds are able to enter the binding site of CYP1A2, interacting with the three key protein residues in a manner similar to previous compounds, with favorable binding energy values of −7.3 and −9.8 kcal/mol for compounds **9** and **26**, respectively. Furthermore, both compounds were able to interact through a hydrogen bond with Ala317. Compound **26** was also found to interact through two π-stacking interactions with Phe125 and Phe226.

### 2.7. Summary of the Assessment of the Binding Affinity

The docking score obtained in our docking experiment was favorable in all cases, and, since it approximately corresponds to the binding free energy of these ligands, their association could be predicted to be in the low micromolar range.

It is difficult to compare our findings with those reported in the literature for a number of reasons. In fact, besides the usual difficulties in assessing binding free energy in the experiment (e.g., ruling out specific effects due to the solvent, salt concentration, choice of the buffer, etc. [46]), an additional difficulty is that for prodrugs, the binding parameters cannot result from interpreting data of a simple association/dissociation reaction taking place under thermodynamic equilibrium. For this reason, binding affinity might be estimated only indirectly. For instance, a constant *K*_m_ in the micromolar range was obtained for 6-MNA, the active metabolite of Nabumetone, by exploiting the Michaelis–Menten kinetics equation in experiments on human liver microsomes [21].

On the other hand, although simulation techniques exist that are more accurate than molecular docking to perform free energy calculations [47], applying them in the present case (i.e., for such a large number of ligands) is impractical. Simpler methods, based on molecular mechanics and generalized Born surface area (MM/GBSA), were previously employed to assess the binding of ligands to CYP1A2 in a number of cases [48,49,50,51,52]. However, binding energies calculated by these methods resulted in predicted binding energies in the low femtomolar range, or sometimes even more favorable. This would suggest a practically irreversible binding, which does not correspond to realistic modeling.

In summary, within the limitations of the molecular docking technique, our results constitute a reasonable assessment of the affinity of compounds to CYP1A2, in a scenario where both experimental complexes (including crystallographic ones) and computational models are still limited.

## 3. Methods

Molecular docking of the studied prodrugs was carried out on the crystallographic structure of CYP1A2 (PDB code 2HI4) [15], in which the enzyme is co-crystallized with BHF [53], following a procedure already adopted for other studies [54]. We had previously used this protocol for other drug discovery and design endeavors [55,56]. The molecular structures of the ligands were built using the molecule editor Avogadro, and their geometry was optimized through energy minimization with the universal force field (UFF) [57]. Preliminary conversion of the structures from the PDB format to PDBQT, in which partial charges are given, was carried out by using the graphical interface AutoDock Tools 1.5.6 [58]. During the conversion, polar hydrogens were added for the crystallographic enzymes, and the apolar hydrogens of the compounds were merged with the carbon atoms they were attached to. Docking calculations were performed by using AutoDock Vina 1.1.2 [59], either exploring the search volume that included the protein structure or by performing a score-only assessment without any search in the case of re-docking of the crystallographic ligand in its known binding pose. In the former case, a search volume of 20 Å × 20 Å × 20 Å centered on the protein pocket left empty after removing the crystallographic ligand was considered, and a very high exhaustiveness of search was used, eight times larger than the default value [60,61]. Full flexibility was guaranteed for the ligands, facilitated by the relatively small number, from 1 to 7, of active torsions around bond dihedral angles, except for Trepibutone (**19**) (11 torsions). All compounds with an acidic nature were tested in the anionic form. The binding modes of the ligands were analyzed through visual inspection, and interaction energies and distances were quantified using Molecular Operating Environment (MOE) 2018.01 (Chemical Computing Group ULC, Montreal, QC, Canada). The Molecular Graphics System PyMOL [62] was used to visualize protein structure and ligand binding using a customized script based on the sole structure of the docking complex. Chemical formulas were created by using ChemDraw [63].

## 4. Conclusions

Arylalkane-derived prodrugs of arylacetic acids endowed with anti-inflammatory activity can be easily prepared by simple acylation of an aromatic portion with a succinic anhydride or by alternative straightforward methods. Known derivatives of this type were subjected to molecular docking experiments with CYP1A2, and all of them proved to be able to interact with the enzyme. The tested compounds exhibited a unique mode of interaction within the active site of CYP1A2. In particular, the alkanoic portion of the molecules enters the heme proximal region, delimited by Ala317 and Thr321, where it can undergo metabolic transformation. On the other hand, the aromatic portion protrudes towards the cavity opposite to the heme, thus favoring the establishment of hydrophobic interaction with Phe226, and is not involved in any substantial modification.

All these observations provide detailed suggestions on the key residues of CYP1A2 involved in the binding and on the molecular determinants that drive the association of ligands to the protein catalytic site. Overall, the results herein reported could help in future rational design endeavors for developing anti-inflammatory agents. In particular, from these findings, valuable suggestions can be drawn that could be exploited by using quantitative structure–activity relationship (QSAR) models or computational tools that may include molecular docking and more advanced computational techniques. Within the limits of the molecular scaffold herein explored, favorable binding energy values were recorded for all the compounds, and drug-enzyme complexes are stabilized by several hydrophobic interactions with key amino acid residues of the catalytic pocket of CYP1A2. These overall results suggest that further alterations on both the aromatic and alkanoic moieties could lead to novel agents being efficiently converted in vivo into more powerful metabolites active in various inflammatory pathologies.

## Data Availability

The datasets generated and/or analyzed during the current study are available from the corresponding authors on reasonable request.
